# 

**Published:** 1887-04

**Authors:** 


					﻿Vol VII.
APRIL, 1887.
NO. 4.
THE
pOMIOPATHlC PHYSICIAN,
A MONTHLY JOURNAL OF MEDICAL SCIENCE.
** He is a freeman whom truth makes free, and all are slaves beside."
"Seek the Truth; come whence it may, cost what it will."
EDITED BY
EDMUND J. LEE, XI. ID.,
AND
WALTER XI. JAMES. XI. L>.
PHILADELPHIA :
No. 1123 SPRUCF: STREET.
2ST E W YORK.
A. L. CHATTERTON & COMPANY, Pdblisher’s Agents.
L O iT D O N-:
ALFRED HEATH A CO., Sole Agents fob Great Britain,
114 Ebury Street, EatOu Square.
Yearly Subscription, $2.50. invariably in advance. Single numbers, 95 cts.
Entered at the Philadelphia Poet-Office as Second*Class Mail Matter.
				

